# Enhancing Resin Cement Adhesion to Zirconia by Oxygen Plasma-Aided Silicatization

**DOI:** 10.3390/ma15165568

**Published:** 2022-08-13

**Authors:** Li-Li Kang, Shu-Fen Chuang, Chia-Ling Li, Jui-Che Lin, Ting-Wen Lai, Ching-Cheng Wang

**Affiliations:** 1 Institute of Manufacturing Information and Systems, National Cheng Kung University, No. 1 Universal Road, Tainan 70101, Taiwan; 2 School of Dentistry, Institute of Oral Medicine, National Cheng Kung University, No. 1 Universal Road, Tainan 70101, Taiwan; 3 Department of Stomatology, National Cheng Kung University Hospital, 138 ShengLi Road, Tainan 70403, Taiwan; 4 Department of Chemical Engineering, National Cheng Kung University, No. 1 Universal Road, Tainan 70101, Taiwan

**Keywords:** 10-methacryloyloxyidecyl-dihyidrogenphosphate (MDP), tribochemical silica-coating, silane, zirconia, bonding, atmospheric pressure oxygen plasma, silicatization

## Abstract

The combinations of alumina particle air abrasion (AA) and a 10-methacryloyloxyidecyl-dihyidrogenphosphate (MDP) primer and a tribochemical silica coating (TSC) and a silane–base primer are contemporary pre-cementation treatments for zirconia restorations for bonding with resin cements. However, the stability of zirconia resists the mechanical or chemical preparations. The purpose of this study was to develop an atmospheric-pressure oxygen plasma (OP)-aided silicatization method to enhance the adhesion of resin cements to zirconia. Zirconia discs were prepared to receive surface treatments of different combinations: (1) AA or TSC (2) with or without OP treatment, and (3) a chemical primer (no primer, silane, or a silane–MDP mixture). The surface morphology, hydrophilicity, and chemical compositions were characterized, and the resin–zirconia bond strengths were examined either after 24 h or a thermocycling test. The results indicated that the OP treatment after the TSC facilitated the homogeneous distribution of silane and crosslinking of silica particles, and effectively improved the hydrophilicity. The OP increased the O and Si and reduced the C elemental contents, while the combination of TSC, OP, and silane induced SiO_x_ generation. Among the groups, only the TSC-OP–silane treatment effectively enhanced the bond strength and maintained the adhesion after thermocycling. With these results, the OP aided the silicatization protocol effectively, generated silane crosslinking, and resulted in superior resin–zirconia bond strength and durability compared to the current treatments.

## 1. Introduction

In recent decades, dental metal–ceramic restorations have been replaced with ceramic systems due to the rising costs of precious metals and the unpleasing opacity of metal copings [1]. Yttria-stabilized tetragonal zirconia polycrystalline (Y-TZP) ceramics, commonly containing 3 mol% yttria, have been widely accepted as promising materials for fabricating dental restorations due to their superior mechanical and aesthetic properties [2]. Y-TZP ceramics exhibit high fracture toughness, which is mainly due to the transformation toughening effect of their tetragonal phase [3]. Zirconia-based tooth- and implant-supported fixed prosthodontic restorations have shown high survival rates, maintaining extreme precision in terms of the margin adaptation [4,5]. Zirconia-based restorations rely on the resin luting cements to remain fixed to the tooth, while zirconia’s chemical stability and densely packed structure indicates its resistance to bonding with resin [6]. Zirconia does not respond to common chemical modifications such as hydrofluoric acid (HF) etching and silanization due to its acid resistance and lack of silica contents [7]. Different strategies have been developed to establish resin–zirconia bonding and to ensure long-term stability. Roughening treatments such as air abrasion with alumina particles and grinding are popularly used techniques, while the generated micro-mechanical retention is insufficient to sustain functional loading [7,8]. Aggressive mechanical treatments also raise concerns due to the generation of critical flaws, which in turn induce premature catastrophic failure [9]. The combination of air abrasion and chemical agents containing organophosphate esters results in significant improvements in adhesion. Among these agents, 10-methacryloyloxyidecyl-dihyidrogenphosphate (MDP) has been proven to bond to zirconia via ionic and covalent bonds and to link with resin cements [10,11,12]. However, the coordinate bond between the MDP and zirconia is vulnerable to hydrolysis and the integrity of the bond interface may degrade after a thermocycling test or long-term immersion [13,14,15].

Silicatization is another approach to increase the surface reactivity of zirconia. Selective infiltration etching was developed based on heat-induced nano-structural changes to allow the diffusion of a specific molten glass into the zirconia. The fused silica phase is subjected to HF etching to be retentive, whereby the adhesive resin monomers can infiltrate and interlock [16]. Other silica coating protocols, including pyrolytical silica coating, plasma spray, vapor-phase deposition, and sol–gel dip-coating techniques, have been introduced in order to modify the zirconia surface [17]. Among them, tribochemical silica coating (TSC) has become the most popular method in view of its convenient handling and evident efficacy [9,13,17]. The TSC technology has been innovated upon since 1989, and has become a favored method utilized in chemically inert materials. The original CoJet-Sand and Rocatec systems (3M ESPE, Seefeld, Germany) are the most popular commercial products for applying this technique. The TSC technique grit blasts the substrates with silica-coated alumina particles, thereby embedding and coating the surfaces with silica caused by the exerted kinetic energy. The TSC method is also referred to as “cold silicalization” because the chemical modification takes place without any change in temperature. Another successor system, SilJet Plus (Danville Inc., Danville, IL, USA), composed of silica-encapsulated silane, has been marketed and has been demonstrated for the silicatization of metal and ceramic surfaces [18]. A silane agent is generally used following the TSC treatment to allow the silanol units to form chemical bonds with the adhered silica, and the methacrylate groups copolymerize with the resin monomers [19]. Compared to other silica coating methods, the TSC provides great retention for zirconia and is less technically complicated [13,18]. However, the resulting silica coating is not uniform, with only a small fraction of zirconia surface coverage [20]. Furthermore, the created silica coating does not strongly adhere to the zirconia surface, meaning the retention decreases overtime [9,21]. Therefore, some investigators still suggest the use of an MDP-based primer after the TSC treatment to achieve durable bonding [8,18,19,22].

Plasma technology has been widely applied in biomedical engineering and is recognized as an economical and effective method for surface modifications [23]. Plasma treatments may enhance the surface reactivity of substrates without changing the desirable bulk properties. Among the various plasma treatments, atmospheric-pressure plasma is more feasible for dental laboratory or chair-side work to improve the resin–zirconia bonding, since it does not require a vacuum chamber or device [24,25]. The selection of the source gases, power, or power parameters affects the action mode and the generated reactive species. Oxygen plasma is excellent for removing organic pollutants, since the active atomic oxygen may interact with hydrocarbon compounds on the surface and cleave them into H_2_O and CO_2_ [26,27]. Based on the evidence from previous studies, atmospheric-pressure oxygen plasma (APOP) can also transfer oxygen-containing polar functional groups onto zirconia surfaces, thereby increasing the oxygen-to-carbon (O/C) ratio in treated zirconia and improving the surface reactivity [24,26,28,29]. Nevertheless, the current experimental results show that the sole atmospheric-pressure plasma treatment did not improve or maintain the long-term stability of resin–zirconia bonding [30,31].

The use of a TSC together with silanization has been a common silicatization approach for adhesion substrates, regardless of the non-uniformity and small fraction of surface coverage [32]. The plasma-aided silicatization treatment may provide another solution for the modification of zirconia surfaces. The use of oxygen plasma (OP) is a common processing method used to clean or etch silicon and silica substrates in the fabrication of integrated circuits. APOP has been particularly applied in the low-temperature deposition of silica film on substrates, analogous to the processing studies on low-pressure plasmas [33]. Accordingly, the purpose of this study was to develop a novel APOP-aided silicatization method to enhance the resin–zirconia bonding. The specific aim was to explore whether APOP after the TSC enhanced the surface silicatization of zirconia for the adhesion of resin cements. Different protocols consisting of grit blasting techniques, oxygen plasma, and priming agents (a silane–base mixture and a silane–MDP mixture) were used for comparison. The hypotheses examined were as follows: (1) there will be no difference in the bond performances of the zirconia samples after different surface treatments; (2) the APOP treatment will not interact with the other surface treatments, in turn affecting the zirconia bonding.

## 2. Materials and Methods

### 2.1. Sample Preparation

**Figure 1:**
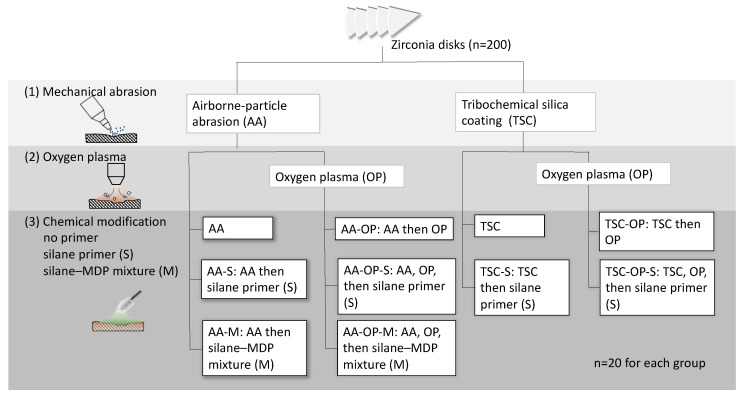
Treatments for the experimental groups.

Cercon zirconia blank cylinders (DeguDent, Hanau, Germany) were cut into disks (19.5 mm in diameter and 2.5 mm in thickness) and divided into eight pieces. A total of 200 pieces were generated and fully sintered. These specimens were polished serially using 320-, 400-, and 600-grit silicon carbide papers. The polished specimens were placed in 95% alcohol, subjected to ultrasonic cleaning for 5 min, and then dried. Afterward, the specimens were divided into ten groups to receive different combinations of surface treatments: (1) grit blasting treatment: alumina particle air abrasion (AA) or TSC, (2) with or without APOP treatment; (3) primer treatment: no primer, with a silane–base or silane–MDP mixture primer. The treatments for the experimental groups are illustrated in Figure 1.

For the AA treatment, the zirconia disks were grit-blasted utilizing 45 µm Al_2_O_3_ particles (WA320, Rich Sou, Kaohsiung, Taiwan) at a distance of 10 mm for 15 s at 2.5 bar pressure. The specimens were ultrasonically cleaned in deionized water three times each for 10 min. Subsequently, they were dried using an oil-free air gun. For the TSC treatment, the zirconia specimens were grit-blasted at the same distance with 30 μm silica-coated Al_2_O_3_ (Rocatec Soft, 3M ESPE, St. Paul, MN, USA) at 2.8 bars for 15 s.

In the 2nd step of the surface treatments, half of the specimens were subjected to APOP treatments using a pen-style atmospheric-pressure oxygen plasma jet (ATM-1 Air Aurora Processor, AP Plasma Co., Taichung, Taiwan). The plasma jet consisted of two concentric cylindrical stainless-steel electrodes. The outer electrode was 18 cm long, with a 25 mm inner diameter and a converging nozzle with a diameter of 2.5 mm. The plasma was generated using an oxygen gas flow at 9.5 L/min, under a pulse power excitation of up to 80 W. The assigned zirconia disks were placed 5 mm under the plasma jet to receive the gas stream for 30 s. During the plasma treatment, the surface temperature of the specimens reached 160 °C. The OP-treated specimens received their assigned primers after 2 s, when their temperature decreased to approximately 80 °C.

The last step of the surface treatment involved the chemical modifications. To investigate the silicatization caused by different agents, the specimens were divided into three subgroups: no primer, a silane–base primer (S), or an experimental primer in the form of a mixture (M) of silane and MDP. For groups AA, AA-OP, TSC, and TSC-OP, no primer was applied. For groups AA-S, AA-OP-S, TSC-S, and TSC-OP-S, the zirconia disks were coated with 12 µL of the silane–base primer Clearfil Porcelain Bond Activator (Kuraray Medical, Okayama, Japan) for 5 s using a micropipette, and dried using the oil-free air gun. For groups AA-M and AA-OP-M, an experimental primer of the MDP–silane mixture was prepared following our previous work [10]. Equal volumes of Clearfil Porcelain Bond Activator and Clearfil SE Bond primer (Kuraray Medical) were dispensed and mixed. Next, 12 µL of the experimental primer was applied on each assigned disk for 5 s, then the disk was then dried.

### 2.2. Surface Micromorphology

Three treated specimens in each group were gold-sputtered and then observed under a low–variable vacuum scanning electron microscope (Inca 350, Oxford, UK). In the preliminary micromorphologic findings, only TSC-S and TSC-OP-S showed the presence of surface coatings. Therefore, the zirconia specimens in these two groups were further examined under a field-emission SEM (Zeiss Auriga, Carl Zeiss, Jena, Germany) at high magnification.

### 2.3. Hydrophilicity Test

To examine the changes in the hydrophilicity of each specimen, 2 µL of deionized water was dispensed on the specimen surfaces as the probing liquid. A charge-coupled device camera was employed to capture the images of the contact angles on the specimen surfaces (*n* = 5). The contact angles were then analyzed using ImageJ software.

### 2.4. X-ray Photoelectron Spectroscopy (XPS) Analysis

The surface element composition and chemical state of the treated specimens in each group were analyzed using X-ray photoelectron spectroscopy (PHI-5000 Versaprobe, ULVAC-PHI Inc., Osaka, Japan). The specimen was placed in a vacuum chamber under 5 × 10^−10^ bar. A monochromatic Al Ka X-ray source operated at 25 kV and a pass energy of 117.40 eV were used to obtain survey scans of the elements in order to assess the atomic percentages. To identify the chemical compositions, fine scans of the O 1s, C 1s, Si 2p, Zr 3d, and P 2p regions were conducted using the X-ray source operated at 25 kV and a pass energy of 23.5 eV. The resolution for the fine scanning was 0.1 eV. A peak deconvolution and quantitative analysis of the chemical compositions was performed using the ULVAC-PHI MultiPak™ software program.

### 2.5. Shear Bond Strength (SBS) Test

The resin–zirconia bonding was examined using early and post-thermocycling shear bond strength (SBS) tests. In each group, twenty zirconia disks were prepared and treated with the assigned treatments. Pre-cured resin composite tablets (3-mm-diameter and 2-mm-thick) were fabricated using a microhybrid resin composite named Filtek Z250 (3M ESPE, St. Paul, MN, USA). An adhesive cellulose acetate tape with a 3 mm hole was attached to the zirconia surface. A dual-cure resin luting cement (Variolink II, Ivoclar Vivadent, Schaan, Liechtenstein) was filled in the hole, and the composite tablet was cemented and loaded under 5 N of force. The excess resin cement was cleaned, and the tape was carefully removed. The cemented specimen was light-cured at 850 mW/cm^2^ with a quartz halogen light unit (Optilux 501, Demetron Kerr, Orange, CA, USA) for 40 s from different directions. The light intensity was verified using a radiometer (Coltolux light meter, Coltene-Whaledent). The specimens were left to set for 30 min and placed in a 37 °C water bath for 24 h. Subsequently, half of the specimens (*n* = 10) in each group received the early (T0) SBS test, and the other half (*n* = 10) were subjected to 6000 thermocycles (T6000). The thermocycles consisted of alternative soaking in 5 °C and 55 °C deionized water baths, with a 30 s dwelling time at each temperature and a 5 s transfer time between the two baths. For the SBS test, the resin–zirconia assemblies were fixed on the jig of a universal material test machine (AGI, Shimadzu, Kyoto, Japan). Force was applied near the bond interface using a chisel at a crosshead speed of 1 mm/min until fracture. After the SBS test, the zirconia specimens were sectioned to inspect the bond interface between the zirconia and the adhesive layers.

### 2.6. Statistical Analysis

The statistical analysis was performed with SPSS 20.0 (IBM Corp., Somers, NY, USA). The contact angles in the wettability test and the SBSs of all experimental groups were analyzed using a one-way analysis of variance (ANOVA) followed by a post hoc Duncan test. An additional three-way ANOVA test was performed to assess the individual effects of the grit blasting, APOP processing, primer agents, and their interactions on the SBSs. An independent samples *t* test was conducted to examine the effects of the thermocycling on the SBSs. The significance level was set at *p* < 0.05.

## 3. Results

### 3.1. Surface Micromorphology

**Figure 2:**
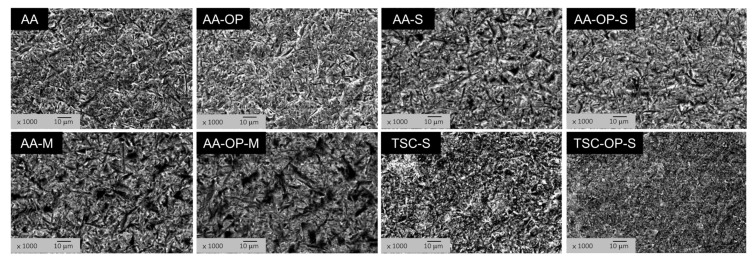
SEM images (1000×) after the surface treatment. Only TSC-OP-S showed an evident homogeneous coating on the grit-blasted surfaces.

Under SEM, the treated zirconia specimens showed roughened surfaces (Figure 2). All AA group specimens exhibited more significant notches and microcracks compared to those in the TSC groups, and showed no plastic deformation. In the AA-M and AA-OP-M groups, the more darkened areas indicated the accumulated primer with low electrical conductivity in these notches. TSC-S and TSC-OP-S showed a homogeneous coating on the surfaces, which was thicker in the latter.

**Figure 3:**
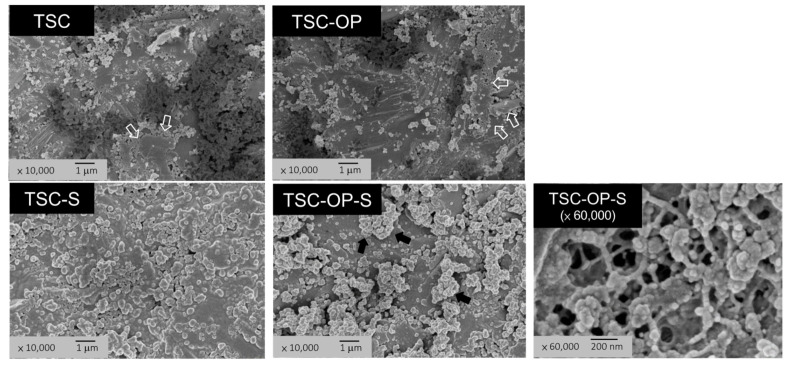
High-magnification images of the TSC-treated specimens (10,000×). TSC and TSC-OP exhibited retained Rocatec soft particles and fused layers (hollow arrows). TSC-OP-S showed a homogeneous coating, while TSC-OP-S showed particle agglomerates (solid arrows) on the coated layer. Under 60,000× magnification, a network structure with linkages of SiO_2_ particles was found in TSC-OP-S.

The micromorphology of the TSC-treated specimens was further examined at higher magnification (10,000×) (Figure 3). TSC-alone illustrated retained Rocatec soft particles and a plastically fused layer that partially covered the zirconia surface. TSC-OP had a similar morphology, except that less particles were retained. Both TSC-S and TSC-OP-S exhibited a coating layer with particle agglomerates, which were more prominent in TSC-OP-S specimens. Under 60,000× magnification, the surface coating of TSC-OP-S presented a network structure with linkages of the SiO_2_ particles.

### 3.2. Hydrophilicity Test

**Figure 4:**
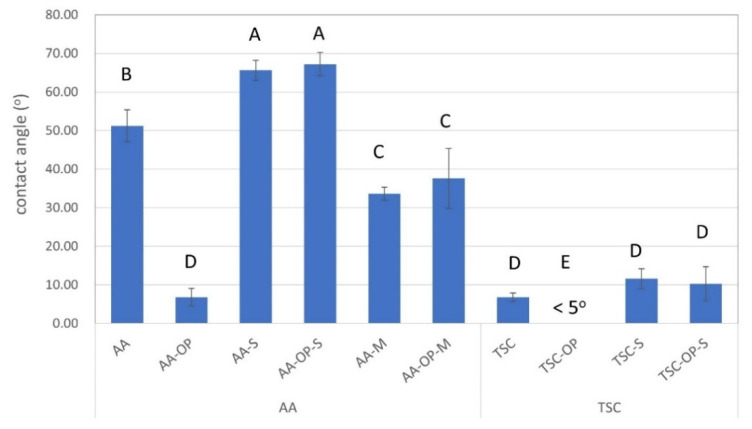
Mean values of the contact angles in all experimental groups. Different upper-case letters indicate a significant difference (*p* < 0.05) among the experimental groups.

The results from the contact angle test identified significant differences among the groups (*p* < 0.05) (Figure 4). AA-alone generated a hydrophobic surface with a mean contact angle of 51.2°, but the following OP significantly reduced the contact angle (6.8°). The silane treatment following AA (AA-S and AA-OP-S) led to extremely hydrophobic surfaces, while the use of the silane–MDP mixture in the corresponding groups (AA-M and AA-OP-M) led to moderate hydrophilicity.

All of the TSC-treated groups showed high hydrophilicity (contact angles less than 12°). The TSC-OP group even showed superhydrophilicity, with unmeasurable contact angles. Similar to AA-S and AA-OP-S, the silane conditioning following TSC also increased the contact angles, but only to a minor degree.

### 3.3. XPS Analysis

**Figure 5:**
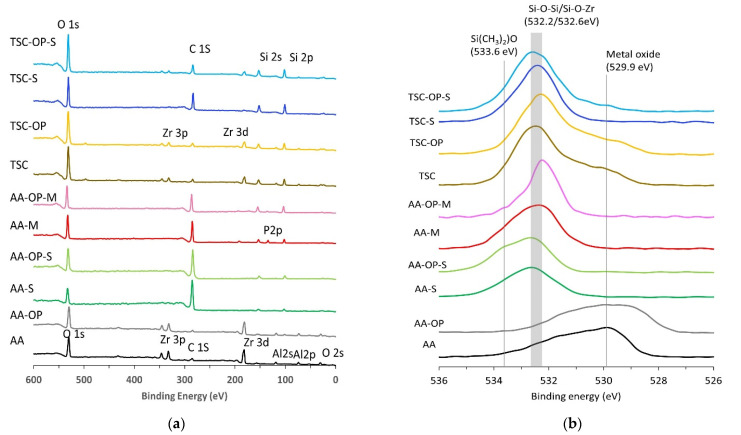
XPS spectra of the experimental groups: (**a**) full spectra; (**b**) O 1s narrow scan spectra.

The XPS spectra of the eight experimental groups are illustrated in Figure 5a. In groups AA, AA-OP, TSC, and TSC-OP, the presence of Zr 3d and Zr 3p peaks indicated the exposed zirconia surface. The Al 2s and Al 2p peaks in the AA and AA-OP groups suggested the presence of retained Al_2_O_3_ particles. Weak P 2p signals were present in AA-M and AA-OP-M.

Fine scanning of the individual O 1s peaks was performed to analyze the chemical state of the treated zirconia (Figure 5b). The O 1s peak centered at 529.9 eV in the AA and AA-OP groups was attributed to the lattice oxygen in the metal oxides [27]. In the primer-coated groups, the primary O 1s peak significantly shifted toward higher binding energy, which indicated the presence of Si-O-Si and Si-O-Zr bonds (532.2 and 532.6 eV, respectively) [28]. AA-S and AA-OP-S showed the presence of a weak peak representing inactivated silane (Si(CH_3_)_2_)O) at 533.6 eV.

The elemental compositions are listed in Table 1. As shown in the full spectra, AA and AA-OP showed the presence of Zr and Al. All TSC-treated groups exhibited high silicon contents (>16%). The groups with OP treatments showed increased O contents but reduced C contents compared to their counterparts without OP, thereby increasing their O/C elemental ratios. The OP treatment also slightly enhanced the Si content.

**Figure 6:**
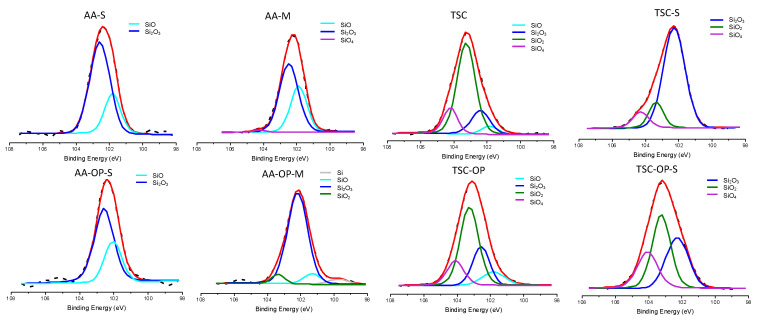
Deconvolution of the Si 2p spectra in the experimental groups.

In the groups using the chemical primers, the Si 2p region was deconvoluted into the Si element (99.8 eV), SiO (101.5 eV), Si_2_O_3_ (102.5 eV), SiO_2_ (103.5 eV), and SiO_4_ (104.1 eV) (Table 2) (Figure 6) [29,30,31]. In all AA-treated groups, Si_2_O_3_ and SiO were the primary and secondary SiO_x_ states. Differing from these groups, SiO_2_ was the primary chemical state of TSC and TSC-OP, which was attributed to the silica coating layer. In the TSC-S group, the Si 2p peaks obviously shifted to Si_2_O_3_, indicating a siloxane layer or linkage of the silane and the silica coating. In the TSC-OP-S group, the SiO_2_ and SiO_4_ replaced Si_2_O_3_ to become the primary chemical state.

### 3.4. SBS Test

In the T0 SBS test, the AA-OP-M and TSC-OP-S groups exhibited higher SBSs, followed by AA-M and TSC-S (Table 3). AA-alone had the lowest SBS value, as the chemical modification of either the OP or a priming agent significantly (*p* < 0.05) improved the bonding strengths. 

After 6000 thermocycles, all AA-alone specimens failed before the test. The AA-OP and AA-S groups also showed low bond strengths (2.6 and 3.3 MPa, respectively), while AA-OP-S exhibited a much higher SBS (16.7 MPa). The *t* test showed that all groups had dramatic reductions in SBSs after thermocycles, with the exception of the AA-OP-S and TSC-OP-S groups. Actually, TSC-OP-S exhibited a comparable SBS to its T0 counterpart.

According to the three-way ANOVA test, either the priming conditions, OP treatment, or thermocycling test substantially (all *p* < 0.001) influenced the SBS at both T0 and T6000 stages. For the T0 SBSs, there were interactions between the mechanical abrasion and OP treatment, as well as between the primer and the OP treatment. In terms of the T6000 SBSs, there was no interaction between any two factors.

**Figure 7:**
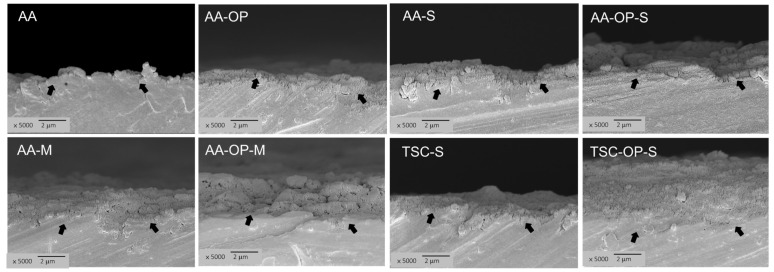
Cross-sectional images of debonded TC6000 zirconia specimens.

With the images of sectioned TC 6000 specimens (Figure 7), AA had the thinnest residual adhesives, which corresponded to the adhesive failure pattern. Groups incorporating OP showed more retained primer or resin cements than their non-OP counterparts. AA-M and AA-OP-M presented thick residual stacks, which were loose and partially detached at the interfaces. The residual primer and cements of TSC-S and TSC-OP-S bonded closely to the zirconia surfaces, while the latter had the thickest and densest adhesive layers, without any evident internal defects.

## 4. Discussion

For surface treatments of zirconia restorations, the combinations of AA and MDP primers and TSC followed by silane–base primers are two recommended protocols. However, the resin–zirconia bonding approach using MDP-based primers or universal adhesives is susceptible to hydrolysis [13], while TSC does not lead to the complete silicatization of zirconia [8,18,20]. In this study, the APOP-aided silicatization was developed via the integration of APOP between the TSC and silane primer treatments. The efficacy of this new method was assessed with regard to the micromorphology, hydrophilicity, chemical modification of zirconia, and promotion of resin–zirconia bonding.

APOP may generate reactive species to attack the hydrocarbon pollutants and turn them into CO_2_ and H_2_O, which can be removed from the surface via the gas flow [27]. In our previous study on porcelain–zirconia bonding, the APOP jet deepened the grain boundaries and enhanced the nano-mechanical interlocking of veneering porcelain [29]. The APOP in this study did not etch the surface of the zirconia, neither did it obviously change the surface morphology. On the other hand, the APOP altered the chemical state of the zirconia surface with the increased oxygen and decreased carbon contents. The intensity of the C-C/C-H bond was also reduced, which indicated the removal of organic contaminants. Zirconia ceramics exhibit adsorption properties related to the CO_2_ on their surfaces [35]. The uptake of CO_2_ in zirconia yields hydrogen and other carbonates, which may impair the wetting of the resin luting cements [11]. The APOP jet in this study provided the cleaning function and promoted the interactions of the chemical primers and resin cements with the zirconia. As seen in the results of the SBS tests, the groups receiving APOP treatments showed higher bond strengths compared with their counterparts. In this regard, the APOP treatment enhanced the adhesion of the resin cements to the zirconia ceramics, irrespective of the grinding and primer treatments.

The APOP treatment also changed the Si contents and states. In this study, the Si content possibly originated from the SiC polishing papers, silica-coated particles, or silane-containing primers. The deconvolutions of the Si 2p spectra showed that the silane agent generated prominent Si_2_O_3_ peaks in the AA-S, AA-OP-S, AA-M, AA-OP-M, and TSC-S groups (Figure 6). The OP treatment led to the shift of the Si 2p peak toward a higher binding energy, indicating an increase in the oxygen-rich components and the O/Si ratio. In TSC-OP-S group, the chemical state of the Si 2p significantly shifted to SiO_2_ and SiO_4_, which suggested the formation of siloxane bonds with γ-MPTS [36,37]. Previous studies have shown that a heat treatment following the application of silane removed the alcohol and reaction byproducts and led to high siloxane bond formation [38,39]. The residual heat brought about by the APOP may also evaporate the solvent and activate the crosslinking of the silane coupling agent. This APOP-aided silicatization was also depicted in the O 1s spectra (Figure 5b). In the primer-coated groups, the shifting of O 1s to the Si-O-Si bond (532.2 eV) was contributed to by the oligomerization of silane or the interlinkage between the silane and coated silica [40]. Despite the low reactivity of zirconia, the formation of rich siloxane groups can induce additional covalent bonds with the underlying zirconia. Similar findings have been reported in an earlier study, in which the Si–O–Si bond and Zr–O–Si bonds co-existed in the silane-modified zirconia powder and were evidenced by FTIR and NMR analyses [41].

In this study, the surface wettability of the treated zirconia was related to all of the processes, including the grit blasting, use of APOP, and chemical primer. For the AA-OP group, the hydrophilicity was enhanced because the plasma transferred reactive species to break down the pollutants on the surface and reduced the interface tension between the water and the specimen [27,42]. On the other hand, the APOP did not affect the AA-OP-S and AA-OP-M groups, since their surface wettability was predominantly determined by the chemical primers. All TSC-treated specimens showed high wettability, and TSC-OP-S was even superhydrophilic. Since the TSC-OP treatment enables surface silicatization, the generated SiO_x_ and existence of Si-O-Si bonds lead to strong reactivity to water.

Since the experimental mixture primer was prepared by dispensing silane and MDP at a 1:1 ratio and applied at the same volume, the Si content of AA-M should be half that of AA-S. However, AA-M showed a higher Si content than AA-S did (Table 1). The acidity of MDP may accelerate the condensation and oligomerization of γ-MPTS, forming an interphase consisting of both chemically and physically adsorbed silane [39,43]. When the heat brought about by APOP evaporated the solvent, the condensation reaction was augmented and more siloxane was retained in the interphase. The significantly increased Si content and changes in Si 2p states were validated by the XPS results (Table 2, Figure 6). The excessive oligomerization of γ-MPTS may result in a thicker interphase containing a higher proportion of physically adsorbed silanes, which eventually weakens the bond durability, as reported in a previous study [39].

In the SBS test, the prefabricated composite tablets were cemented with a dual-cure cement (Variolink II) and light-cured immediately after sitting. A previous study revealed that the light activation of the dual-cure cement resulted in better bonding performance compared to the self-curing mode [44]. Even with this design, all of the specimens in group AA failed during the thermocycles. Combining AA with either APOP (AA-OP) or the silane primer (AA-S) improved the initial SBSs at intermediate degrees, but did not maintain the SBSs after thermocycling. Although AA-M and AA-OP-M showed high T0 SBSs (20.6 and 26.5 MPa, respectively), their T6000 SBSs dropped substantially. The MDP–base primer–adhesive has been advocated as the most effective chemical agent to link resin cements and zirconia. The MDP monomers bond to zirconia via the P-O-Zr bond and work as coupling agents to copolymerize with the resin matrix [10,45]. However, the adhesion in this way degrades upon aging or thermocycling due to the lower hydrolytically stability of the MDP [13,15]. The chemical interaction between γ-MPTS and MDP may also affect the effectiveness of the MDP. Recently, MDP-based universal adhesives have been developed to be applied in pre-cementation treatments of ceramic restorations. Some of these universal adhesives comprise both silane and MDP to cover a variety of dental ceramics, including silica-based and zirconia ceramics. In our previous work using a time-of-flight secondary ion mass spectrometry (ToF-SIMS) analysis, the co-existing silane increased the hydroxylation of zirconia and weakened the adhesion of resin cements [10]. Our recent study further validated that the addition of silane into MDP-based universal adhesives impaired the adsorption of MDP to zirconia [11]. In this study, we found that both the MDP and APOP accelerated the oligomerization of silane. The combination of the APOP and MDP further induced a thicker silane interphase in the adhesive layers, which was vulnerable to hydrolysis. The evidence was shown in the cross-sections of failed AA-M and AA-OP-M specimens, where the adhesive layers exhibited internal defects (Figure 7).

Among the groups, only TSC-OP-S effectively enhanced and sustained the SBSs after thermocycling. The OP-aided silicatization method has multiple benefits in enhancing the resin cement’s adhesion to zirconia. As the TSC generated a partially fused silica coating on the surface, the APOP gas flow removed the loose silica coatings but left the bonded ones. The heat melted the coatings and transformed them into dense clusters or tightly silicate layers (Figure 3; TSC-OP-S under 60,000×). The heat also evaporated the solvent of the silane primer and accelerated the crosslinking reaction of γ-MPS with the silicate layers. The generated Si-O-Si bonding may be strong and stable to resist hydrolysis, since there was no detectable leakage at the bond interface, even after thermocycling (Figure 7). These processes result in strengthened silicatization on the zirconia surface, and promote the adhesion of resin cements.

With the above findings, the first hypothesis was rejected. The type of grit blasting, use of APOP, and use of a chemical primer all affect the bond performance. The second hypothesis was also rejected, since the APOP had a synergistic effect on improving the adhesion when combined with the TSC or a silane agent, but had an antagonist effect when combined with the MDP. Among the groups, the protocol using the TSC then APOP and silane lead to strong resin–zirconia bonding and favorable durability. The incorporation of the APOP between traditional TSC–silane treatments facilitates surface silicatization, which could be an effective pre-cementation treatment for dental zirconia restorations to establish reliable adhesion with resin cements. With the advances in plasma technology, various micro-plasma jet systems can be applied for this purpose. In this regard, this study still presents limitations, since various AP conditions were not examined. Future research about the source gas and operating parameters may provide in-depth information about the plasma species concentration, and the species’ effects on the surface modifications and mechanical properties of the zirconia. Additionally, this study did not assess the combination of a TSC, APOP, and silane–MDP mixture. The interpolation between the primers and APOP-assisted silicatization should be explored for further improvement of the adhesion of resin cement to zirconia restorations.

## 5. Conclusions

This study developed a new silicatization method for dental zirconia restorations via serial treatments of a TSC, APOP, and a silane coupling agent. This method avoids the problems of adhesion hydrolysis associated with AA-M treatments and incomplete silica coatings caused by TSC. The reactive oxygen species generated by the APOP may clean the organic pollutant and loosen the silica coating, and the residual heat strengthens the crosslinking of the silica coating and silane. Accordingly, an intact bond interface is formed, which leads to strong and durable resin–zirconia bonding. With the initial findings, the incorporation of APOP with AA or a silane–MDP mixture primer may enhance the initial bond strength, but the APOP has no effect on the performance against thermocycling. In this regard, the APOP is not recommended to be combined with MDP-based or universal adhesives.

## Figures and Tables

**Table 1:** XPS analysis revealing the element compositions (%) on the surfaces of the experimental groups.

Group	Zr	Y	Al	Si	P	O	C	O/C
AA	10.6	0.9	11.3	1.2	-	58.1	17.8	3.3
AA-OP	9.5	0.5	12.9	4.1	-	65.5	7.5	8.7
AA-S	0.1	-	-	3.2	-	16.3	80.4	0.2
AA-OP-S	-	-	-	4.1	-	24.4	71.5	0.3
AA-M	-	-	-	8.7	3.2	25.6	60.9	0.4
AA-OP-M	-	-	-	13.1	2.1	34.9	49.8	0.7
TSC	3.5	4.6	-	16.8	-	62.4	12.7	4.9
TSC-OP	3.1	5.4	-	17.6	-	55.5	18.5	3.0
TSC-S	0.5	-	-	17.7	-	33.0	48.8	0.7
TSC-OP-S	1.5	-	2.3	18.5	-	47.5	30.2	1.6

**Table 2:** Area percentages (%) of different chemical states in the deconvoluted Si 2p peaks.

Group	Si ^1^	SiO ^1^	Si_2_O_3_ ^1^	SiO_2_ ^1^	SiO_4_ ^2^
AA-S		27.7	72.3		
AA-OP-S		30.9	69.1		
AA-M		36.2	62.4		1.4
AA-OP-M	6.5	11.8	74.6	7.1	10.2
TSC		5.5	17.4	62.8	14.3
TSC-OP		11.0	22.3	51.1	15.6
TSC-S			75.7	14.1	10.2
TSC-OP-S			33.6	42	24.4

^1^ Si: 99.8 eV; SiO: 101.5 eV; Si_2_O_3_: 102.5 eV; SiO_2_: 103.5 eV [29]. ^2^ SiO_4_: 104.1 eV [34].

**Table 3:** The mean (standard deviation) SBS values.

Group	x				Significant Difference between Non-OP and OP	
	Nil		OP			
	T0	T6000	T0	T6000	T0	T6000
AA-x	6.2 (1.5) ^Aa^	0 (0) ^Ab^	13.3 (3.5) ^Ba^	2.6 (0.9) ^Bb^	*	*
AA-x-S	11.5 (1.8) ^Ba^	3.3 (1.9) ^Bb^	17.3 (1.8) ^Ca^	16.7 (3.1) ^Da^	*	*
AA-x-M	20.6 (1.7) ^Da^	13.3 (1.2) ^Cb^	26.5 (4.0) ^Ea^	14.7 (2.0) ^Cb^	*	
TSC-x-S	19.8 (2.0) ^Da^	17.1 (2.3) ^Db^	25.8 (2.5) ^Ea^	24.2 (3.4) ^Ea^	*	*

Different upper-case letters indicate a significant difference (*p* < 0.05) among the experimental groups at either the T0 or T6000 stage. Different lower-case letters in the same group indicate a significant difference (*p* < 0.05) between the SBSs at the T0 and T6000 stages. Note: * indicates a significant difference (*p* < 0.05) in the comparisons between non-OP and OP groups at either the T0 or T6000 stage.

## Data Availability

The data presented in this study are available on request from the corresponding author.
